# Left-Handers Are Less Lateralized Than Right-Handers for Both Left and Right Hemispheric Functions

**DOI:** 10.1093/cercor/bhab048

**Published:** 2021-04-22

**Authors:** Leah T Johnstone, Emma M Karlsson, David P Carey

**Affiliations:** School of Psychology, Perception, Action and Memory Research Group, Bangor Imaging Group, Bangor University, Bangor, LL59 2AS, UK; Sport Psychology Group, UCFB, Manchester, M11 3FF, UK; School of Psychology, Perception, Action and Memory Research Group, Bangor Imaging Group, Bangor University, Bangor, LL59 2AS, UK; School of Psychology, Perception, Action and Memory Research Group, Bangor Imaging Group, Bangor University, Bangor, LL59 2AS, UK

**Keywords:** brain asymmetry, fMRI, handedness, left hemisphere, right hemisphere

## Abstract

Many neuroscientific techniques have revealed that more left- than right-handers will have unusual cerebral asymmetries for language. After the original emphasis on frequency in the aphasia and epilepsy literatures, most neuropsychology, and neuroimaging efforts rely on estimates of central tendency to compare these two handedness groups on any given measure of asymmetry. The inevitable reduction in mean lateralization in the left-handed group is often postulated as being due to reversed asymmetry in a small subset of them, but it could also be due to a reduced asymmetry in many of the left-handers. These two possibilities have hugely different theoretical interpretations. Using functional magnetic resonance imaging localizer paradigms, we matched left- and right-handers for hemispheric dominance across four functions (verbal fluency, face perception, body perception, and scene perception). We then compared the degree of dominance between the two handedness groups for each of these four measures, conducting *t*-tests on the mean laterality indices. The results demonstrate that left-handers with typical cerebral asymmetries are less lateralized for language, faces, and bodies than their right-handed counterparts. These results are difficult to reconcile with current theories of language asymmetry or of handedness.

Humans have asymmetrical brains (Hugdahl 2005; Hugdahl and Westerhausen 2010; Porac 2015). The two hemispheres have functional differences, and these asymmetries may lead to more efficient processing and cerebral organization (Corballis 2017). Since pioneering investigations by Broca, Wernicke, Dax, Lichteim, and others in the late 19th century, the association between left-hemisphere lesions and language abnormalities in right-handers became thoroughly documented (Bogen and Bogen 1976; Critchley 1970; Tesak and Code 2008). Given this early prominence of language in early behavioral neurology, a large proportion of the research on brain asymmetry has concentrated on the left hemisphere’s dominance for speech perception and production (e.g., Hickok and Poeppel 2007; Hugdahl and Westerhausen 2010; Vigneau et al. 2006).

Despite evidence that there are no differences in structural cerebral asymmetries between handedness groups (Guadalupe et al. 2014), there are well-established links between handedness and functional cerebral asymmetry for language. Early accounts suggested that the left-handed people would be right-hemisphere dominant for speech and language (Hecaen and Sauguet 1971; Hughlings-Jackson 1880). This sensible hypothesis has turned out to be untrue; in fact, almost 70% of left-handers are left-hemisphere dominant for speech and language (Carey and Johnstone 2014). This unusual characteristic of the majority of left-handers was largely forgotten, in part perhaps because of suggestions of subtle pathology in left-handers—an idea now largely discredited. As left-handers only make up approximately 10% of the population (Papadatou-Pastou et al. 2020), research on language lateralization has traditionally excluded those participants, despite their likelihood to be left-hemisphere dominant (Willems et al. 2014).

Specializations that are thought to favor the right hemisphere have not been given the same attention as language asymmetry. This gap is in part due to a small number of early behavioral studies, which suggested reduced right-hemispheric bias in left-handers for face and spatial processing (e.g., Levy et al. 1983; Bryden et al. 1983). Indeed, much of the literature centered on handedness from behavior (e.g., Bless et al. 2015; Kertesz et al. 1992), electrophysiology (e.g., Dundas et al. 2015; Reid and Serrien 2012), and a limited number of imaging experiments (Powell et al. 2012; Willems et al. 2009) support reduced average asymmetry in left-handed groups relative to right-handed controls. This reduced asymmetry in left-handers is found so routinely that most people no longer bother to look. In fact, the face validity of such findings is so convincing that it is likely to have resulted in publication biases that favor findings with such reductions (see for example, Karlsson et al. 2019). The often implicit, assumption is that these right-hemisphere functions, such as processing faces or emotional prosody in speech, are allocated by some causal mechanism to the non-speech/language half of the brain (Bryden 1982; Behrmann and Plaut 2015; Dehaene et al. 2015).

One result of the expectation of asymmetry reduction, or more variability in asymmetry, in left-handers, is their general exclusion from much electrophysiology and neuroimaging work related to language and speech processing (Bailey et al. 2019; Willems et al. 2014). Reduced asymmetries on average, however, may disguise a more nuanced picture in the actual data itself*.* It could result from most left-handers having identical cerebral dominance to right-handers, but with some individuals having reversed dominance. Of course, a weaker mean bias is just as plausibly accounted for by reduced asymmetries in left-handers *en masse*, independent of the hemisphere which is dominant. These two distinct causes of reduced average asymmetry have dramatically distinct theoretical implications (Karlsson et al. 2019). The often-implicit assumption in laterality studies follows the first argument: a reduced mean asymmetry in the left-handers is the result of the small proportion showing a reversed lateralization, with the majority being lateralized in both direction and degree as the right-handers. There is no obvious reason why the second argument is not just as credible: that many of the left-handers are typically lateralized, but to a lesser extent than their right-handed counterparts.

The underlying cause of reduced average asymmetry in left-handed groups is easily tested, but rarely ever carried out. Two obvious approaches are worthy of consideration. The first is to focus on estimates of the frequency of left typical and atypical cerebral asymmetries (particularly non-language ones) in right- and left-handed groups (Carey and Johnstone 2014; Karlsson et al. 2019).

The second approach, which is pursued here, is to ensure that the handedness groups or subgroups are directly comparable with one another and then compare the characteristics of the measured asymmetry. The over-representation of people with right hemisphere or bilateral language dominance in the left-handed group means that any comparison of typical dominance averages as a function of handedness is confounded. Instead, the most telling contrast is handedness, but within right or left dominance groups. For example, an important unasked question for language asymmetry is whether the 70% of left-handers with left hemispheric dominance are as lateralized as right-handers with left hemispheric dominance (about 95% of them). This important contrast has yet to be made for language, let alone with any other asymmetries, such as those that favor the right hemisphere.

Here, we used functional magnetic resonance imaging (fMRI) to measure four different cerebral asymmetries (language, face perception, body perception, and scene perception) in the same 58 left-handers and 33 right-handers. We quantified asymmetries in individual people using a robust and reliable (Johnstone et al. 2020) technique that does not depend on an arbitrary statistical threshold decided on for an entire group (Wilke and Lidzba 2007). Because individuals are classified as left- or right-dominant, we can control for the confounding effects of more individuals with the rare atypical asymmetry (which is potentially more common in left-handers) on any overall estimate of hemispheric specialization. Therefore, we investigated averages for the typical pattern of hemispheric lateralization (left-hemisphere dominant for verbal fluency, right-hemisphere dominant for faces, bodies and scenes). We hypothesize that removing the confound of heterogeneous left-handed groups in terms of cerebral dominance should result in either:

- no difference between the right-handed and left-handed participants, or
- a remaining (albeit more difficult to account for) reduced mean asymmetry in the left handers.

Only then can an unbiased estimate of magnitude of asymmetry for individuals who show the typical bias be generated.

## Materials and Methods

### Participants

In total, 93 participants took part in this experiment—33 right-handed (21 female) and 58 left-handed (22 female). Participants were recruited via opportunistic sampling, but with a particular emphasis on recruitment of the rarer, left-handers. Including a higher number of these is necessary to ensure a sufficient sample with typical lateralization of functions (estimated as 70% of the sample). Two participants (both left-handed, one male and one female) were excluded from the analysis due to excessive head movements (>4 mm). Right-handed subjects had a mean age of 26.09 (SD = 5.92) and a mean Waterloo handedness questionnaire (WHQ; Steenhuis and Bryden 1989) scores of +28.00 (SD = 2.06). Left-handed subjects had a mean age of 24.83 (SD = 7.55) and a mean WHQ of −20.38 (SD = 13.31). Sex differences in handedness are reliable across samples, with a modest increase in likelihood of left-handedness in males (12%) versus females (10%; see Papadatou-Pastou et al. 2008). Although unequal numbers of males and females across groups may not be desirable, it is also not uncommon in experimental samples. Our dataset is publicly available on the Open Science Framework (https://osf.io/u9f75/) where the full breakdown of data by sex is available. This study received ethical approval from Bangor University Ethics Committee and informed consent was obtained from all participants. Participants were debriefed in detail and offered individual feedback and brain images.

### Language Localizer

A verbal fluency style paradigm was employed. Both an active and a control condition were used in a blocked design. A total of 14 active and 14 control blocks were alternated with 30 rest blocks, each with a duration of 15 s. In the active blocks, participants were presented with a single letter in the middle of the screen for the duration of the block. During this time, participants were instructed to silently think of as many words as they could which begin with that letter. A practice phase was run outside the scanner using the letter “D”. In the control blocks, participants were shown either the letter string “RARA” or “LALA,” and were instructed to mentally repeat these non-words for as long as they were presented on the screen. In the 30 rest blocks a fixation cross was presented and participants were instructed to relax. This task is based on that used by Van der Haegen et al. (2011). The 14 letters chosen were the letters that begin the most words in English: T, A, S, H, W, I, O, B, M, F, C, L, D and P (as reported in the Natural Language Toolkit 3.0—http://www.nltk.org/). This task was presented across two runs, comprising seven active/control blocks per run. The letters were randomly presented in any order across these two runs.

### Face/Body/Scene Localizer

A four-condition localizer was used to identify any asymmetry in face-, body-, and scene-selective brain activation. The task involved viewing blocks of images from the categories: faces, bodies, chairs, and scenes. Although viewing the stimuli, participants completed a simple one-back task, pressing a button if they saw a consecutive, repeated image. This task is based on localizers used in Downing et al. (2006) and Harry et al. (2016). The hand in which participants held the button box was counterbalanced within the right-handed and left-handed groups. Each localizer run consisted of 16 active blocks (4 for each stimulus category) and 5 rest blocks (taking place in block 1, 6, 11, 16, and 21). Each block lasted 16 s during which 16 images were displayed for 300 ms followed by a blank screen for 700 ms. Participants completed two runs of this task, with two different fixed stimulus orders, which were counterbalanced across participants, separately for the right-handed and left-handed groups.

## MRI Acquisition

All scans were acquired in a Philips 3 T Achieva magnetic resonance scanner, using a 32-channel head coil, located at the Bangor Imaging Unit at Bangor University. T1-weighted structural images were obtained with the following parameters: TR = 12 ms, TE = 3.5 ms, FA = 8°, field of view (FOV, mm) = 240 × 240, acquisition matrix = 80 × 79; 175 contiguous slices were acquired, voxel size (mm) = 1 × 1 × 2 (reconstructed voxel size = 1 mm^3^). Functional images were acquired with the following parameters: a T2-weighted gradient-echo EPI sequence; FOV = 220 × 220, acquisition matrix = 96 × 96, 36 slices were acquired; acquired voxel size (mm) = 2.3 × 2.3 × 2.5 (reconstructed voxel size [mm] = 2.3 × 2.3 × 2.5). Verbal fluency (repetition time [TR] = 2500 ms, echo time [TE] = 30 ms, flip angle (FA) = 90°) consisted of two runs of 174 volumes, and the four-condition localizer (TR = 2000 ms, TE = 30 ms, FA = 90°) consisted of two runs of 166 volumes. The first five scans of each functional run were discarded before image acquisition to establish steady-state magnetization.

### MRI Processing

All MRI data were pre-processed and analyzed using SPM12 (Wellcome Department of Cognitive Neurology, University College London, http://www.fil.ion.ucl.ac.uk/spm/) implemented in MATLAB R2015b 8.6 (Mathworks Inc., Sherborn, MA, USA). Anatomical images were first manually aligned to the anterior and posterior commissure (AC-PC). Pre-processing of functional scans consisted of corrections for head motion (spatial realignment; trilinear interpolation), and images were realigned to the first functional volume of the first session (the volume closest to the anatomical scan). Functional scans were coregistered to their corresponding individual anatomical scans and normalized to standard MNI space (3-mm isotropic voxels). Normalized data were then spatially smoothed using a Gaussian kernel of 6-mm full-width at half-maximum. The general linear model was used to map the hemodynamic response curve onto each experimental condition using boxcar regressors. This boxcar function was then fitted to the time series at each voxel resulting in a weighted beta-image. The fitted model was converted to a t-statistic image, comprising the statistical parametric map.

### Statistical Analysis

To assess hemispheric contribution for processing a particular stimulus type, the LI-toolbox plugin for SPM was used (Wilke and Lidzba 2007; Wilke and Schmithorst 2006). This toolbox provides an estimate of how lateralized a participant is for a given contrast by calculating a laterality index (LI) value for each individual contrast. LI values range from −1 (exclusively right hemispheric) to +1 (exclusively left hemispheric). Whole-brain LIs were calculated for each person and task using the following contrasts: faces *>* scenes, bodies *>* chairs, and scenes > chairs. A whole brain analysis with the cerebellum excluded was carried out for fluency *>* letter string, as cerebellar involvement in language processing is contralateral to the activation of the cerebral cortex (Jansen et al. 2005).

Participants were first classified as right hemispheric (LI < 0) or left hemispheric (LI > 0) for each of the four tasks. Only participants with typical (i.e., left hemisphere dominance for verbal fluency and right hemisphere dominance for faces, bodies, and scenes) dominance for each of the task, independent of their dominance for the other tasks were included for the average analysis. IBM SPSS Statistics for Macintosh (Version 25.0. Armonk, NY: IBM Corp.) was used to calculate the mean and standard error for each task by handedness group. One-tailed t-tests were used to compare the mean LIs for the two handedness groups for fluency, faces, bodies, and scenes respectively, using an alpha level of 0.05.

A second analysis comparing average asymmetries for faces, bodies and scenes respectively, was also carried out. This analysis was to ensure that the reduced asymmetries for these three right hemisphere functions were not driven by individuals who were right hemisphere dominant for language. In this analysis, individuals who were right hemisphere dominant for verbal fluency were excluded, and *t*-tests were carried out to compare the two handedness groups.

## Results

**Figure 1:**
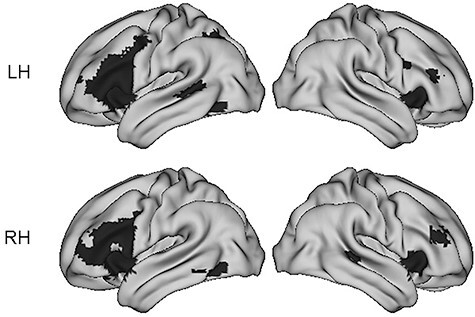
Threshold-dependent group activation maps for individuals left lateralized for verbal fluency, as a function of handedness. (LH = 43; RH = 31). The data are visualized at a threshold of *P* < 0.001 with family-wise error (FWE) correction at the cluster level.

**Figure 2:**
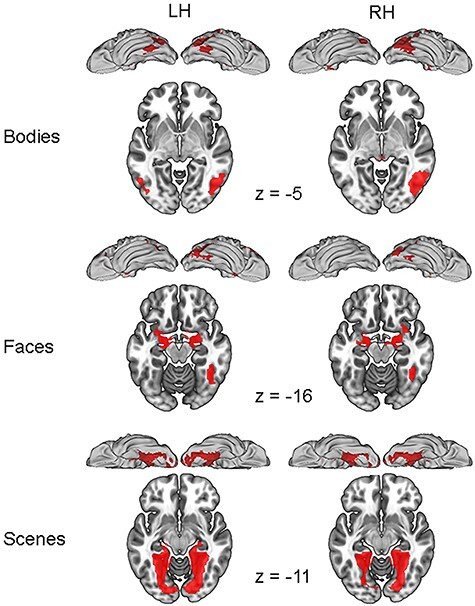
Threshold-dependent group activation maps for individuals right lateralized for bodies (top row), faces (middle row), and scenes (25; bottom row) as a function of handedness. The data are visualized at a threshold of *P* < 0.001 with FWE-correction at the cluster level.

**Figure 3:**
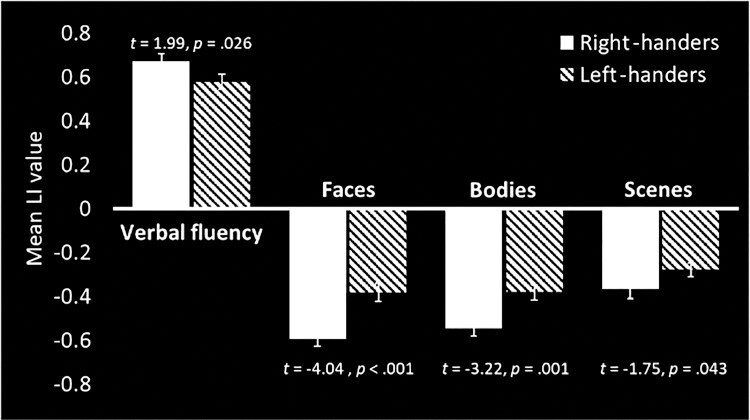
Mean laterality index (LI) scores for the four functions, only in individuals who show typical dominance for each. Error bars indicate standard error. LI values > 0 represent threshold-independent left-hemisphere dominance. The mean LI asymmetry is reduced in all four left-handed samples. Note that the bars for the four different asymmetries were derived from slightly different individuals, as the only inclusion criteria for each was typical dominance for that function. All *P* values are one-tailed.

Figure 1 (verbal fluency) and Figure 2 (bodies, faces, and scenes) show threshold-dependent group activation maps for the right-handed and left-handed participants. Figure 3 and Table 1 show the average threshold-independent laterality indices, with standard errors, as a function of handedness group. As mentioned above, inclusion criterion for the four elements of this analysis was typical dominance (i.e., left-hemisphere dominance for verbal fluency and right-hemisphere dominance for faces, bodies, and scenes). As Figure 3 shows, the left-handed participants have significantly lower LIs than the right-handers for all four asymmetries tested.

**Table 1:** Participant numbers, LI statistics (mean and standard deviations), *t*-values, and effect sizes (Cohen’s d) from *t*-tests for each function and group. LIs calculated on a scale from −1 (exclusive right hemisphere activation) to +1 (exclusive left hemisphere activation).

	*N*	Mean	SD	*t*	*d*
Fluency					
RH	31	.67	.18	1.99^*^	0.48
LH	43	.58	.22		
Faces					
RH	25	-.59	.16	-4.04^***^	1.03
LH	34	-.38	.24		
Bodies					
RH	31	-.54	.20	-3.22^**^	0.78
LH	38	-.38	.21		
Scenes					
RH	25	-.37	.21	-1.75^*^	0.46
LH	33	-.28	.18		

^*^ *P* < 0.05, ^**^P < 0.01, ^***^P < 0.001.

**Figure 4:**
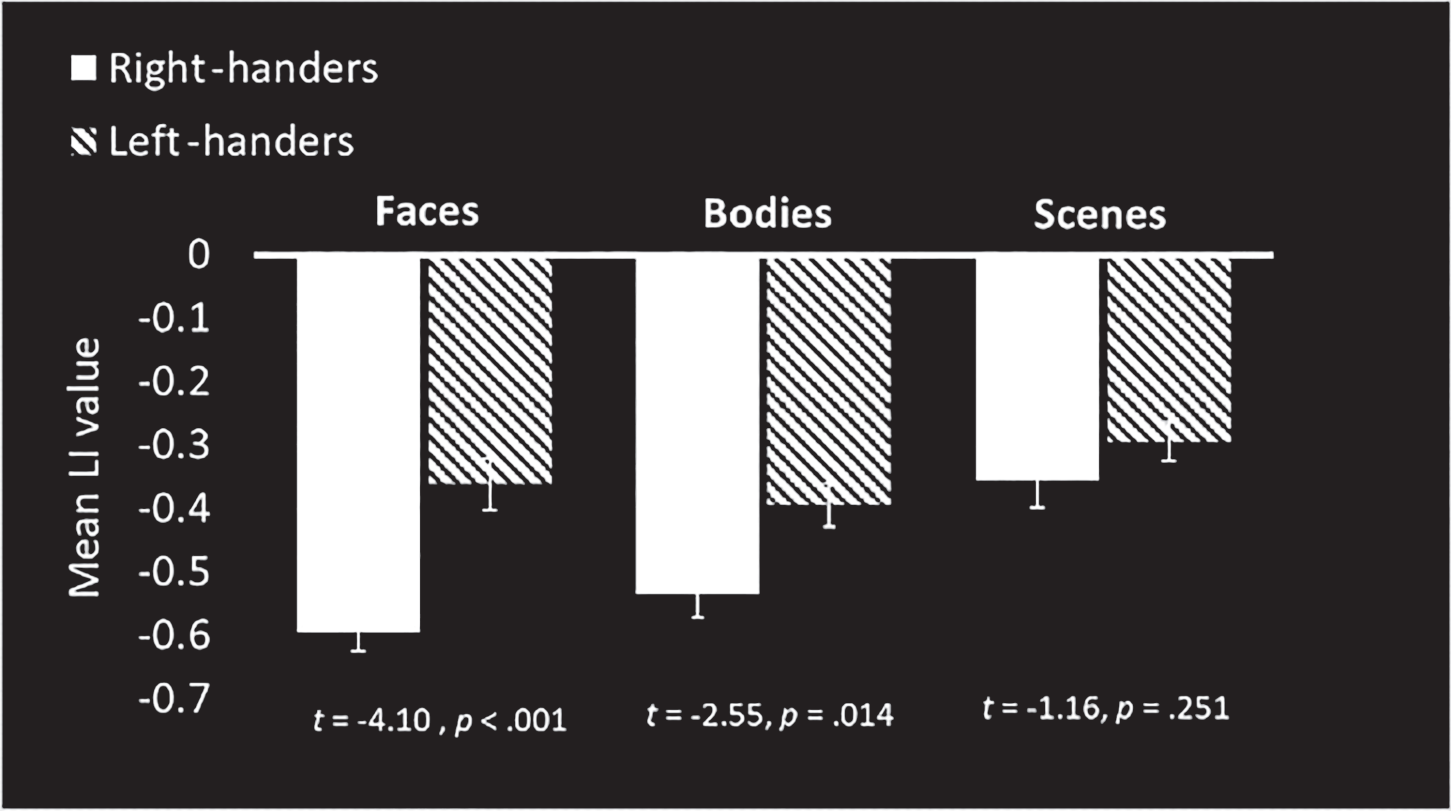
Mean laterality index (LI) scores for the three non-language functions, only in individuals who show typical dominance for each, without the left-handed language atypicals (who happened to be typical on these functions). Error bars indicate standard error. The mean LIs remain significantly reduced in the left-handers for face and body processing.

Controlling for all the other asymmetries within each function (e.g., face dominance within the estimates for body dominance) would be admirable, but would require an even larger sample. Nevertheless, to assess whether the group differences for the three right hemispheric functions could be in part driven by reduced bias in the left-handed individuals with atypical (right) language asymmetry, these participants were removed. Despite the decreases in sample size (a reduction of *n* = 7, 9, and 3 for faces, bodies and scenes, respectively), removing them does not change the pattern, although the difference between groups is no longer statistically significant for right-hemispheric scene perception (Fig. 4).

## Discussion

Decreased asymmetries in left-handers within typical dominance groups for at least three of our four asymmetries are indeed curious. For verbal fluency, the decrease cannot be driven by atypical language dominance because such individuals, by definition, were not included in the calculation. The decrease in the mean right-hemispheric bias in left-handers, for faces and bodies, cannot be explained by inclusion of atypical language dominance either, given our exclusion of these individuals in the secondary analysis.

Stronger asymmetries in right-handers are invariably found in experiments of any sort (Bryden 1982; Carey and Johnstone 2014; Karlsson et al. 2019). None of them, to the best of our knowledge, control for the (potentially) increased proportions of atypical dominance in the left-handed group in the way done here. In fact, with this confound removed, the reductions in typical dominance magnitude are puzzling, indeed. If they are not driven by increased numbers of individuals with atypical language asymmetry in left-handers, there are remarkably few models that could account for them. For example, explanations based on experiential consequences of left-handedness, such as living in a right-handed world (Westmoreland 2017), seem fanciful as a decent model of reduced right-hemispheric bias for, for example, face and body perception.

The only other likely possibility would follow from genetic models of handedness that suggest more varied patterns of asymmetry in some left-handed people (e.g., McManus 1999; Annett 2002). These models postulate a subset of such people, whose genotype results in random localization of different functions to one hemisphere or the other. Excessive co-localization of certain asymmetries could lead to crowding, which might lessen their magnitudes favoring the dominant hemisphere. For example, functions that share similar circuitry within a hemisphere, such as reading and face/body perception (Behrmann and Plaut 2015; Dehaene et al. 2015; Centanni et al. 2018) might each be less lateralized if they share circuitry and dominance with the hemisphere that is specialized for reading. We suggest that certain classes of asymmetry of this sort could be the exception to completely random development of asymmetry, particularly if functions have different developmental time courses.

If models suggesting random allocation of asymmetric functions in some left-handers are correct, individuals who do lateralize randomly might be more easily identifiable, phenotypically, at least, if multiple cerebral asymmetries are measured and quantified on an individual basis in large numbers of left-handers. This kind of large sample size initiative is more likely now, given better sharing by neuroimaging groups interested in asymmetry as part of the general trend to more open, transparent science. Classifying individuals in them as typical or atypical for hemispheric specialization in question should be an important consideration for such studies in future.

This approach could be beneficial in attempts to find structural or functional correlates of either brain asymmetry or handedness (or both). Many approaches have tried to find morphological markers of handedness or cerebral asymmetry (or the interaction of them both), but with modest success to date. For example, left-sided bias in the planum temporale (Dos Santos Sequeira et al. 2006; Tzourio-Mazoyer and Mazoyer 2017) or differences in the shape and distribution of fibers in cerebral commissures such as the corpus callosum (Moffatt et al. 1998; Westerhausen et al. 2006; Haberling et al. 2012; Cowell and Gurd 2018) have been touted as biomarkers many times in the past. These attempts usually have modest success at best, even in more recent variants where larger sample sizes have been thrown at the structure or structures in question (e.g., Guadalupe et al. 2014; Tzourio-Mazoyer and Mazoyer 2017). If the structures are related to language (or some complementary function such as face processing) these efforts will need large sample sizes as effects will be small: most left-handers are left-hemisphere dominant just like most right-handers. Nevertheless, approaches that quantify functional or structural connectivity might prove to be more fruitful with sub-classifying handedness groups by the brain dominance measure of interest (see Labache et al. (2020) for an example of this approach). Clearly something different is happening in the brains of the majority of left-handers that has to do with atypical control of the oral versus manual musculature relative to most right-handers, and left-handers with right hemispheric language dominance (Kimura 1993). This latter type of approach will inevitably be cleaned up to a degree by being able to group individuals by the relevant cerebral asymmetry, although attempts of this sort have not improved the story for the planum temporale in humans (Tzourio-Mazoyer et al. 2018).

Another enterprise might also benefit from classifying participants in the way advocated here. These experiments typically try to demonstrate relationships between behavioral performance on some language-related measure and a measured cerebral asymmetry (e.g., Tzourio-Mazoyer et al. 2018) Modest correlations are typically obtained, but correlational analyses are inevitably elevated by having different latent groups in the datasets. Predictions about cerebral asymmetries should work within typical and atypical dominance groups, and not just between them. These efforts, however well motivated, contain often unknown reliabilities related to both the behavioral and the neuroimaging measurement (although see Johnstone et al. 2020). Additionally, it is difficult to know a priori if either measure is the optimal one for estimating relationships between functional asymmetries and behaviors. For example, there are many ways to localize face-selective regions, and the obtained lateralization indices of these approaches clearly differ (for example, compare our approach here versus Badzakova-Trajkov et al. 2010). There is no single consensus technique for quantifying language laterality using fMRI (fluency, used here, works well if the goal is to produce well established proportions of typical and atypical dominance for handedness (Johnstone et al. 2020). There are good reasons to suspect that different tasks will produce quite dramatically different estimates of language lateralization in the same individuals (Woodhead et al. 2019, 2021).

The historical focus on speech/language asymmetry and the left hemisphere in neurology and neuropsychology is understandable, given the centrality of language, handedness and motor skill in many models of hominid evolution. The data presented here suggest reduced hemispheric specialization in left-handers for verbal fluency, as well as body and face processing, in left-handers with typical hemispheric lateralization. These latter reductions in asymmetry are particular puzzling as they remain even when confounding effects of atypical language dominance are removed from the equation. It may be time to have a second look at the so-called minor hemisphere, in left-handed people in particular.

## Funding

The Leverhulme Trust (grant RPG-2019-102 to D.C.); Bangor University (125 Anniversary Scholarship to L.J.); Bangor University (School of Psychology PhD Scholarship to E.K.).

## Notes

Bronson Harry, Paul Mullins, Paul Downing, Patricia Bestelmeyer, and Kami Koldewyn provided support and advice on neuroimaging. *Conflict of Interest:* None declared.

## Data Availability

The dataset generated during this study is available on OSF, https://osf.io/u9f75/.
